# Aprovechar la pandemia como una oportunidad para promover los conocimientos sobre las vacunas y la resiliencia frente a la información errónea^*^

**DOI:** 10.26633/RPSP.2021.50

**Published:** 2021-05-12

**Authors:** Robin C. Vanderpool, Anna Gaysynsky, Wen-Ying Sylvia Chou

**Affiliations:** 1 Instituto Nacional del Cáncer Rockville Estados Unidos de América Instituto Nacional del Cáncer, Rockville, Estados Unidos de América.; 2 ICF Next Rockville Estados Unidos de América ICF Next, Rockville, Estados Unidos de América.

La vacunación contra las enfermedades infecciosas ha sido reconocida como uno de los “diez mayores logros de la salud pública” del siglo XX, dado su gran impacto a nivel mundial sobre varias enfermedades, como la poliomielitis, la gripe, la neumonía, el sarampión, la parotiditis, la rubéola, las hepatitis virales, la tos ferina y la infección por el virus del papiloma humano oncógeno (1). Los programas de inmunización han logrado una reducción significativa de los nuevos casos de enfermedad y de la morbilidad y mortalidad asociadas, menores costos de atención de salud y una mejora de la productividad (1). Sin embargo, a pesar de su demostrada efectividad clínica y en cuanto a sus costos, la vacunación aún no ha alcanzado todo su potencial. Las tasas de inmunización en niños y adultos siguen siendo insuficientes, lo que ha llevado al resurgimiento de ciertas enfermedades infecciosas (p. ej., el sarampión).

Poner de relieve la vacunación es especialmente importante ahora que en todo el mundo se combate la COVID-19. Esta nueva enfermedad infecciosa fácilmente transmisible presenta un curso de enfermedad poco conocido, afecta de manera desproporcionada a las personas mayores y a las minorías étnicas y raciales, se presta fácilmente a la información errónea y ha sido politizada. La población, llena de incertidumbre, espera el desarrollo de una vacuna contra la COVID-19 que devuelva a la sociedad a una situación parecida a la normalidad. Por ese motivo, sentar las bases para la aceptación de la vacuna es una prioridad urgente de salud pública. Ahora que la vida cotidiana se encuentra interrumpida y el debate sobre las vacunas domina los titulares de las noticias, las sesiones de gobierno y las redes sociales, debe aprovecharse la oportunidad para educar sobre las vacunas, responder a la reticencia y fortalecer la resiliencia frente a la información errónea sobre las vacunas contra la COVID-19 en particular y sobre la vacunación en general. Para ello, será necesario renovar la colaboración de la población, los líderes comunitarios, los prestadores de atención de salud, los profesionales de la salud pública, los responsables de las políticas y los organismos de salud para abordar los desafíos asociados con el fortalecimiento de los conocimientos, actitudes y comportamientos respecto a las vacunas.

La falta de acceso a las vacunas es un factor clave en las tasas bajas de inmunización en muchas comunidades; eso es un hecho innegable. Sin embargo, cuando el acceso no es un problema, el principal obstáculo para la aceptación de las vacunas es la falta de confianza. De hecho, las últimas encuestas muestran que muchos estadounidenses no tienen intenciones de vacunarse contra la COVID-19 cuando haya alguna vacuna disponible. Por esa razón nos centramos específicamente en las estrategias y líneas de investigación para abordar la reticencia y la educación en materia de vacunas.

Dada la velocidad a la que se están produciendo las vacunas contra la COVID-19, recabar información adecuada sobre su desarrollo y administración, así como sobre su seguridad y eficacia, puede ser difícil para el público general. La confianza en la seguridad y la efectividad de la vacuna podría aumentarse mediante esfuerzos de comunicación proactivos y coordinados (por ejemplo, campañas de concientización pública) que destaquen las fases del desarrollo de las vacunas, la supervisión de la Administración de Alimentos y Medicamentos (FDA) y los sistemas de notificación de eventos adversos (2).

Además de aumentar el nivel de conocimiento sobre las vacunas, los esfuerzos de concientización deberían fortalecer la capacidad del público de evaluar la información de salud de forma crítica, analizar datos numéricos y apreciar la complejidad de la investigación científica (3). Las estrategias de educación sobre las vacunas podrían incluir comunicación personalizada entre proveedores y pacientes (y padres) durante encuentros en entornos clínicos, campañas específicas en los medios de comunicación, sesiones entre pares, cursos sobre salud y ciencia en las escuelas, y programas educativos llevados a cabo en la comunidad (p. ej., en iglesias, en programas de extensión o a través de servicios sociales) (4).

Si bien aumentar el nivel de conocimiento general sobre las vacunas será fundamental, el mayor desafío para los esfuerzos de educación sobre las vacunas posiblemente sea la proliferación de información errónea sobre las vacunas en Internet. Durante años, los expertos en salud pública y defensores de las vacunas han tratado de revertir el daño causado por informes sobre una correlación infundada entre el autismo y las vacunas infantiles. Estos esfuerzos de rectificación se han visto socavados por la enorme cantidad de información errónea que circula en Internet acerca de las vacunas. La información errónea se ha convertido en un problema especialmente grave en el contexto de la COVID-19, ya que todavía son muchas las preguntas sin respuesta sobre la enfermedad. Ante la ausencia de certeza científica, los rumores se afianzan con mayor facilidad.

Resulta alarmante la manera en que los grupos antivacunas aprovechan esta situación para engañar al público general de una manera activa y transmitir por los medios sociales mensajes divisivos, incluso antes del desarrollo de una vacuna contra la COVID-19. Como se indica en diversos informes recientes de los medios, estos activistas usan los medios sociales para propagar un discurso sobre las libertades individuales, anticiparse a cualquier posible ley de vacunación contra la COVID-19, amplificar el miedo y la desconfianza hacia las vacunas, desacreditar a los involucrados en el desarrollo de vacunas (p. ej., compañías farmacéuticas, filántropos, investigadores del gobierno), y para alentar a los padres a no asistir a las citas rutinarias de vacunación durante la pandemia. Estos esfuerzos de difundir desinformación e información errónea en línea son especialmente preocupantes, ya que la investigación muestra una disminución en el número de niños que reciben la vacunación de rutina desde que la COVID-19 fue declarada una emergencia nacional (5).

Cabe destacar que la exposición a la información errónea no puede rectificarse simplemente por medio de iniciativas de comprobación de los hechos, corrección o desacreditación: un amplio acervo de investigación muestra que las retractaciones rara vez influyen sobre la confianza en la información errónea debido al fenómeno de la “influencia continua”. Para mitigar los efectos de la exposición a información errónea sobre las vacunas se debe formular y poner a prueba estrategias novedosas más allá de las iniciativas tradicionales de concientización. Estas estrategias podrían basarse en esfuerzos pasados para desacreditar a la industria tabacalera, como sembrar escepticismo sobre los agentes que difunden información errónea y desarrollar herramientas para ayudar al público a identificar fuentes de información creíbles (p. ej., diseñar un símbolo que indique que una cuenta en las redes sociales o un sitio web es creíble y ha sido comprobado).

Hay otras ideas innovadoras como luchar contra las teorías conspirativas al asociarse con personas que solían pertenecer a grupos que creen en ellas. Estas personas pueden ofrecer información sobre las creencias del grupo y transmitir información de salud basada en la evidencia a los miembros del grupo. Otra estrategia podría consistir en movilizar a la mayoría de la población que está a favor de las vacunas para contrarrestar la información errónea en línea y así mitigar la exposición a dicha información. Las redes sociales también pueden asumir un papel activo y monitorear, marcar y eliminar contenidos o cuentas que promuevan información de salud perjudicial, así como reconfigurar algunas funciones de la plataforma que ayudan a propagar la información errónea.

Si bien es necesario educar sobre las vacunas, abordar la información errónea y fortalecer los conocimientos en materia de salud, esto no es suficiente para responder a la reticencia y mejorar la aceptación de las vacunas. También hay factores cognitivos, emocionales, sociales, culturales y contextuales que influyen sobre las actitudes y comportamientos generales respecto a las vacunas. Algunos ejemplos de estos factores son la ideología política, las creencias religiosas, los silos de información en línea dominados por puntos de vista homogéneos, la desconfianza en la medicina, las posturas sobre la participación del gobierno en las decisiones de salud individuales y la percepción del riesgo de enfermedad. Por eso los esfuerzos de concientización sobre las vacunas deben reconocer que la reticencia no siempre se debe a la falta de conocimiento y deben abordar estos factores al crear y divulgar mensajes que estén en consonancia con los valores individuales, que reconozcan sus inquietudes y que destaquen los beneficios económicos y de salud que presenta la vacunación para las personas, sus familias y sus comunidades.

Las estrategias que posiblemente sean más efectivas incluyen el refuerzo de las normas sociales que promueven la salud, involucrar a los principales formadores de opinión y personas influyentes en los medios sociales en la defensa de las vacunas, y garantizar la formulación de recomendaciones firmes y coherentes por parte de los prestadores de atención de salud. Ahora bien, reconocemos que muchas de estas estrategias de intervención presentan limitaciones (por ejemplo, una efectividad limitada en personas con creencias arraigadas, alcance insuficiente, etc.); por lo tanto, los esfuerzos de comunicación deben combinarse con enfoques basados en políticas como los requisitos de inmunización en escuelas y lugares de trabajo, de manera que la vacunación sea la opción predeterminada para las personas y familias, y así desincentivar el rechazo de la vacunación.

Mientras enfrentamos las consecuencias para la salud pública de la pandemia de COVID-19, es necesario elaborar, poner a prueba e implementar de manera proactiva y reflexiva intervenciones de comunicación oportunas para aumentar la confianza en las vacunas. Incluso en condiciones normales resulta difícil promover la vacunación, pero esto se torna especialmente importante de cara a las futuras vacunas contra la COVID-19, en un contexto actual caracterizado por la polémica y la información errónea generalizada. Revertir los avances logrados contra las enfermedades prevenibles mediante la vacunación no es una opción; la vacunación sistemática de niños y adultos no puede verse comprometida ni retrasada. Debemos aprovechar la atención que se está prestando a la pandemia de COVID-19 y al debate predominante sobre las vacunas como una oportunidad para explorar nuevas estrategias de intervención y reforzar nuestro compromiso con la educación sobre las vacunas y la protección del público general.

## Declaración.

Las opiniones expresadas por los autores son exclusivamente suyas, y el contenido de este artículo no debe interpretarse como la postura oficial del Departamento de Salud y Servicios Humanos, los Institutos Nacionales de Salud o el Instituto Nacional del Cáncer de Estados Unidos, ni refleja necesariamente los criterios ni la política de la *Revista Panamericana de Salud Pública / Pan American Journal of Public Health* o de la Organización Panamericana de la Salud.
